# Iron in Cardiovascular Disease: Challenges and Potentials

**DOI:** 10.3389/fcvm.2021.707138

**Published:** 2021-11-30

**Authors:** Shizhen Li, Xiangyu Zhang

**Affiliations:** Department of Geriatrics, The Second Xiangya Hospital, Central South University, Changsha, China

**Keywords:** iron, cardiovascular disease, iron overload, ferroptosis, iron chelation

## Abstract

Iron is essential for many biological processes. Inadequate or excess amount of body iron can result in various pathological consequences. The pathological roles of iron in cardiovascular disease (CVD) have been intensively studied for decades. Convincing data demonstrated a detrimental effect of iron deficiency in patients with heart failure and pulmonary arterial hypertension, but it remains unclear for the pathological roles of iron in other cardiovascular diseases. Meanwhile, ferroptosis is an iron-dependent cell death that is distinct from apoptosis, necroptosis, and other types of cell death. Ferroptosis has been reported in several CVDs, namely, cardiomyopathy, atherosclerotic cardiovascular disease, and myocardial ischemia/reperfusion injury. Iron chelation therapy seems to be an available strategy to ameliorate iron overload-related disorders. It is still a challenge to accurately clarify the pathological roles of iron in CVD and search for effective medical intervention. In this review, we aim to summarize the pathological roles of iron in CVD, and especially highlight the potential mechanism of ferroptosis in these diseases.

## Introduction

Iron is an essential mineral nutrient involved in numerous biologic processes, namely, heme synthesis, iron-dependent catalytic reaction, DNA synthesis, and mitochondrial respiration (1). Iron metabolism is complex and has received much attention. Iron homeostasis is maintained by elaborate mechanisms involving iron consumption, uptake, transfer, and storage (1). Inappropriate iron overload or deficiency correlates to a wide range of cardiovascular diseases (CVDs). Iron deficiency can impair cardiomyocyte mitochondrial function and energy supplement, leading to cardiac dysfunction (2). An excess amount of iron can also be toxic by producing hydroxyl radicals *via* the Haber–Weiss–Fenton reactions, causing oxidative damage to cellular components like lipids, proteins, and DNA (3). Moreover, iron-mediated cell death, namely, ferroptosis, has recently been reported to induce cardiomyocyte damage and plays an important role in CVD (4).

In 1981, Jerome Sullivan proposed the iron hypothesis to explain the sex differences in the risk of heart disease and the lower incidence of CVD in premenopausal women (5). Since then, it has been investigated for decades to untangle the connection of iron–heart diseases. Numerous epidemiological studies have indicated that body iron levels are associated with CVD. To date, it is definite that iron deficiency is prevalent in patients with heart failure (HF) or pulmonary arterial hypertension (PAH), and intravenous iron supplements can improve the quality of life of these patients and reduce the associated risk of hospitalization (2, 6). However, the clinical observation regarding the iron status and atherosclerotic cardiovascular disease (ASCVD) is still controversial despite some studies supporting that elevated iron store positively correlates with the incidence of coronary artery disease (CAD). Besides, iron status in other CVD remains unclear and it is a challenge to clarify their relation.

The precise mechanisms of iron homeostasis in the cardiovascular system are distinct and complicated. In this review, we aim to summarize the pathological roles of iron in CVD, and especially highlight the potential mechanism of ferroptosis in these diseases.

## Iron Homeostatic Regulation: the Basics

### Systemic Iron Homeostasis

The adult body contains about 3–5 g of total iron, with two-thirds in the form of hemoglobin and myoglobin. The rest of iron is almost bound to ferritin, a specialized cytoplasmic iron storage protein. Only about 0.1% of total body iron constitutes extracellular iron. Normally, senescent or damaged erythrocytes are phagocytized by macrophages in the spleen and other organs and release their iron into circulation, which can be recycled for heme synthesis in the bone marrow (7). Except for cyclic utilization of erythrocyte iron, dietary iron intake is an important way for the iron supply to replenish body iron loss *via* gut mucosa (8).

Dietary iron is absorbed by gut mucosa cells *via* two distinct mechanisms based on heme and inorganic forms of iron (9). The heme form of iron is absorbed in the apical membrane of epithelial cells *via* a specific heme transporter, heme carrier protein 1 (HCP1). The heme can be degraded by heme oxygenase-1 (HO-1) to release ferrous iron (Fe^2+^), carbon monoxide, and biliverdin. The absorption of inorganic ferric ion (Fe^3+^) requires two key steps: the conversion of insoluble Fe^3+^ to absorbable Fe^2+^ by cytochrome b reductase 1 (DCYTB) and following transportation of Fe^2+^ by divalent metal transporter protein 1 (DMT-1) across the membrane. Internalized Fe^2+^ enters the cytosolic labile iron pool (LIP). Extra iron can be stored as ferritin or exported through the basolateral membrane by ferroportin (FPN), the only known iron export protein. The exported Fe^2+^ is undergone re-oxidation to Fe^3+^ by membrane-bound hephaestin and binds to transferrin (Tf) for long-distance delivery. Circulating Tf-bound iron can be internalized into the cells of peripheral tissues *via* binding to its receptor, transferrin receptor 1 (TfR1) (10).

Circulating iron levels are predominantly regulated by the transmembrane protein FPN. Hepcidin is a peptide hormone released mainly by the liver, effectively preventing cellular iron efflux *via* promoting FPN internalization and degradation. When hepcidin is transcriptionally downregulated in the condition of enhanced erythropoiesis or iron deficiency, more iron is released into circulation from intestinal epithelial cells, macrophages, and hepatocytes (7). High levels of serum iron and chronic inflammatory states can lead to increased levels of hepcidin. The hepcidin-FPN axis tightly regulates systemic iron homeostasis to meet body requirements (7).

### Cellular Iron Metabolism

**Figure 1:**
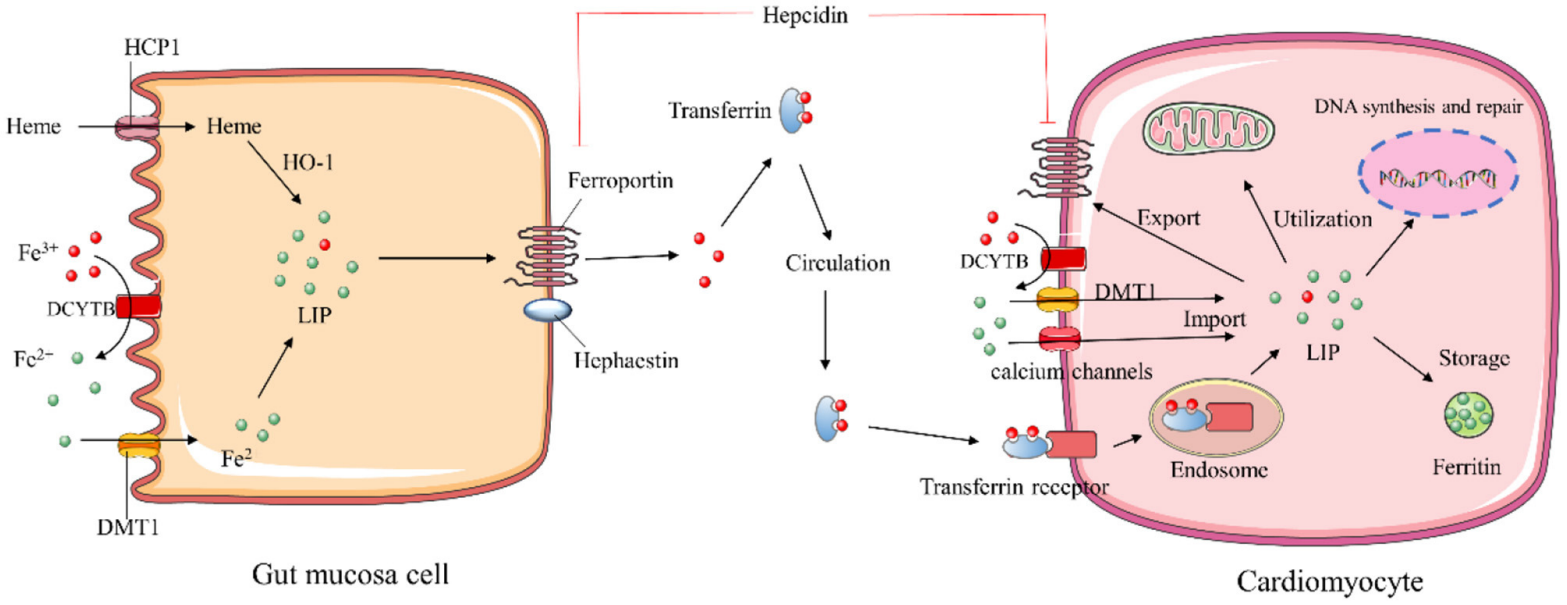
Schematic description of iron homeostasis. HCP1, heme carrier protein 1; HO-1, heme oxygenase 1; DMT-1, divalent metal transporter protein 1; DCYTB, cytochrome b reductase; LIP, labile iron pool.

Iron homeostasis in the body is regulated at both systemic and cellular levels. The Tf-bound iron binds to TfR1 and is internalized by endocytosis, while the uptake of non-Tf-bound iron (NTBI) is mediated by DMT-1 protein ubiquitously present on the surface of cells (11, 12). In addition, the voltage-gated calcium channels of cardiomyocytes can also be iron transporters for NTBI under iron overload conditions (13). After absorption, iron enters the redox-active LIP, where it is utilized for storage in ferritin, or incorporation into iron-require proteins, or trafficking to mitochondria for the synthesis of heme and iron-sulfur (Fe-S) clusters (14) (Figure 1).

Cellular iron homeostasis is post-transcriptionally regulated by the iron regulatory proteins (IRP1 and IRP2) interacting with iron-responsive elements (IREs) (11). IREs are highly conserved hairpin structures of mRNAs present in 5′ or 3′ untranslated regions (UTRs) of iron metabolism genes. IRPs inhibit the initiation of translation by binding to the single 5′-UTR IREs of ferritin and FPN, whereas their binding to the multiple IRE motifs within the 3′-UTR of TFR1 and DMT1 prevents mRNAs degradation (15). The capability of IRPs binding to IRE depends on intracellular iron concentration. In iron-replete cells, IRP1 ligates the Fe-S cluster and functions as a cytosolic aconitase, which precludes IRP-IRE interaction to increase ferritin and TfR1 proteins. In the low intracellular iron environment, IRPs stabilize the mRNA of TfR1 and DMT-1 to enhance iron uptake and inhibit iron excretion by suppressing the translation of FPN (16, 17).

The hepcidin-FPN1 axis also plays a pivotal role in controlling cellular iron flux, particularly in cardiomyocytes. Distinct from systemic iron regulation, hepcidin can be produced locally in the heart, and functions as an autocrine protein to regulate iron levels in cardiomyocytes (18). Interestingly, cardiac hepcidin is upregulated in response to hypoxia to retain cellular iron, while systemic hepcidin is downregulated (19). Such regulation may be an adaptive mechanism to maintain cardiac function.

## Iron and Ferroptosis

### Iron Metabolism and Ferroptosis

Ferroptosis is a novel form of regulated cell death driven by iron-dependent lipid peroxidation. It is distinct from other types of regulated cell death (Table 1), which can be suppressed by iron chelators (e.g., deferoxamine) (20). In the process of ferroptosis, reactive oxygen species (ROS) are overproduced by accumulated intracellular iron, and extensive oxidation of polyunsaturated fatty acid is triggered, resulting in the damage of cellular membrane structure and cell death (20). Thus, modulation of iron metabolism-related genes may regulate ferroptosis by affecting cellular iron homeostasis. The nuclear receptor coactivator 4 (NCOA4) is a selective cargo receptor for the autophagic degradation of ferritin, namely, ferritinophagy, which can increase intracellular iron and induce ferroptotic cell death (21). Overexpression of ferritin heavy chain 1 impaired ferritinophagy and inhibited ferroptosis in PC-12 cells (22). Senescent cells with impaired ferritinophagy were more resistant to ferroptosis (23). Moreover, blockade of cellular iron export *via* genetically deleting FPN has been reported to develop morphological and molecular features of ferroptosis in hippocampus neurons (24). The overexpression of FPN in neurons could alleviate neuronal apoptosis and ferroptosis after intracerebral hemorrhage (25). Taken together, the modulation of cellular iron metabolism might provide a novel therapeutic target for ferroptosis-associated disease.

**Table 1:** Hallmarks of major types of regulated cell death.

**Type**	**Morphological changes**	**Cellular events**	**Major regulators**	**Trigger signals**
Ferroptosis	Mitochondrial shrinkage and increased mitochondrial membrane density	Iron accumulation; lipid peroxidation; ROS accumulation	Positive: TFRC, LOXs, ACSL4, LPCAT3, ALOX15, GLS2, NCOA4, VDAC2/3, RAS, NOX, TfR1, TP53, GLS2s, BECN1 Negative: GPX4, FSP1, HSPB1/5, SLC7A11, NFS1	Iron overload, GSH depletion
Apoptosis	Cell shrinkage, membrane blebbing, chromatin condensation and DNA fragmentation, formation of apoptotic bodies	Phosphatidylserine exposure; DNA fragment; Caspase activation; mitochondria transmembrane potential dissipation	Positive: initiator caspase (CASP2/8/910); effector caspase (CASP3/6/7); pro-apoptotic BCL2 family; TP53 Negative: anti-apoptotic BCL2 family	Death receptor activation
Autophagy	Double-membraned autolysosomes formation	LC3-I to LC3-II conversion; increased autophagic flux and lysosomal activity	Positive:ATG5/7, BECN1 and AMPK Negative: mTOR	Impaired organelles, oxidative stress
Pyroptosis	Lack of cell swelling, rupture of plasma membrane and unaffected mitochondrial integrity	Activation of CASP1 and GSDMD; GSDMDN–induced pore formation; IL-1β release	Positive: CASP1, CASP11, and GSDMD Negative: GPX4, ESCRT-III, PKA	Pathogenic microorganism infection, external stimuli
Necroptosis	Plasma membrane rupture, moderate chromatin condensation and cell swelling	RIPK1, RIPK3 and MLKL phosphorylation; DAMPs release	Positive: RIPK1/3, MLKL	Activation of TNF superfamily receptors

### Regulatory Pathways of Ferroptosis

**Figure 2:**
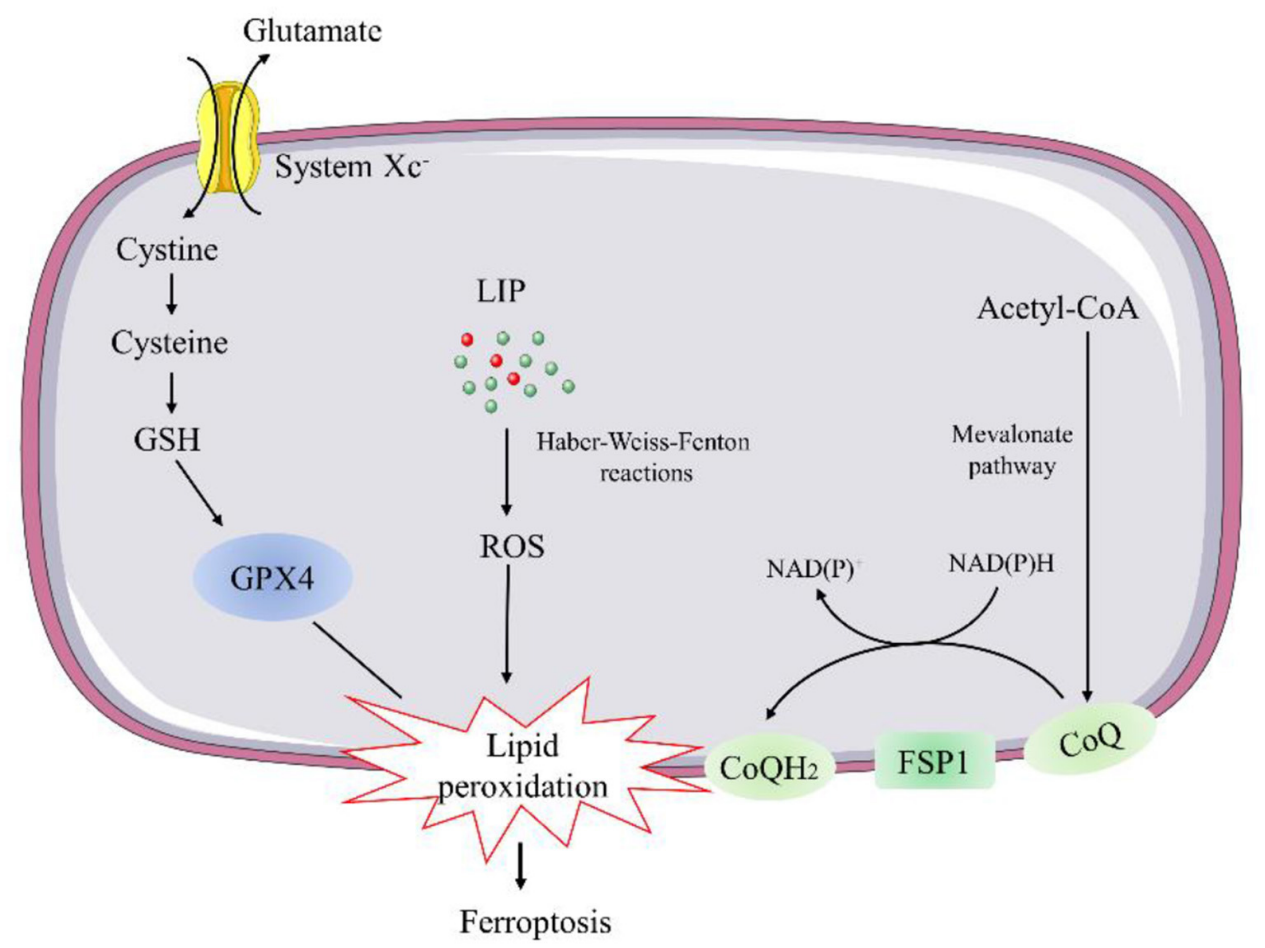
Schematic description of regulatory pathways in ferroptosis. GSH, glutathione; GPX4, glutathione peroxidase 4; LIP, labile iron pool; FSP1, ferroptosis suppressor protein 1; CoQ, coenzyme Q.

Current knowledge indicates two major pathways, the glutathione peroxidase 4 (GPX4)-glutathione (GSH) and ferroptosis suppressor protein 1 (FSP1)-coenzyme Q (CoQ) pathways, involved in the regulation of ferroptosis (26, 27) (Figure 2).

Glutathione peroxidase 4 is a selenocysteine-containing, GSH-dependent enzyme capable of catalyzing the reduction of lipid hydroperoxides (26). Genetic manipulation studies revealed that constitutive deletion or inactive mutant of GPX4 leads to early embryonic lethality. As a result, conditional GPX4 knockout mice were generated to study the mechanisms of GPX4 deficiency-induced cell death. The inducible ablation of GPX4 causes mitochondrial damage and lipid peroxidation-mediated ferroptosis event (28). Thus, several ferroptosis inducers, such as Ras-selective lethal 3, can trigger the accumulation of lipid hydroperoxides and result in cell death by directly inhibiting GPX4 (29). Conversely, overexpression of GPX4 has been reported to protect against oxidative injury in various cell types (30). *ApoE*^−/−^ mice overexpressing GPX4 showed decreased oxidized lipids and atherosclerotic lesions in the aorta compared with *ApoE*^−/−^ control mice (31). Overexpression of mitochondrial GPX4 can also protect ischemia/reperfusion (I/R)-induced cardiac injury (32).

GSH is synthesized from glutamate, cysteine, and glycine in two steps under the catalysis of the cytosolic enzymes glutamate-cysteine ligase and glutathione synthetase, participating in the regulation of ferroptosis (33). Cysteine is the most limiting amino acid for GSH synthesis. Inhibiting its import through the system Xc^−^ is sufficient to trigger ferroptosis *in vitro* (34). System Xc^−^ is a cystine/glutamate antiporter that facilitates the exchange of cystine and glutamate across the plasma membrane (34). Thus, inhibition of system Xc^−^ can lead to deprivation of cellular GSH and impair the function of GPX4 to suppress lipid peroxidation and ferroptosis.

Recent evidence indicates that the FSP1-CoQ10 pathway co-operates with GPX4 and GSH to suppress phospholipid peroxidation and ferroptosis, as a stand-alone parallel system. FSP1, also called apoptosis-inducing factor mitochondrial 2 (AIFM2), was predicted to induce apoptosis by a caspase-1 independent pathway, due to its biochemical similarities to AIFM1 (35). However, FSP1 is recruited to the plasma membrane by myristoylation, where it functions as an oxidoreductase that catalyzes the regeneration coenzyme Q10 (CoQ10) (35), instead of inducing apoptosis. Ubiquinol is the reduced form of CoQ10 generated by the mevalonate pathway. It can act as a lipophilic radical-trapping antioxidant to regulate ferroptosis by halting the propagation of lipid peroxides (35).

### Ferroptosis and Cardiomyopathy

Although the physiological action of ferroptosis remains elusive, it has been studied in several cardiomyopathies, namely, diabetic cardiomyopathy and doxorubicin (DOX)-induced cardiotoxicity.

Diabetic cardiomyopathy, characterized by cardiac hypertrophy, diastolic dysfunction, and intracellular lipid accumulation, is the common complication of diabetes (36). It has been reported that GPX4 expression was reduced in both high glucose-treated cardiomyocytes and the left ventricular myocardial tissues of *db/db* mice (37). A recent study found that inhibition of cardiac autophagy could activate nuclear factor E2-related factor 2 (Nrf2)-mediated ferroptosis and lead to myocardial damage in type 1 diabetic mice (38).

DOX is a second-generation anthracycline chemotherapeutic drug used in many malignancies. It often causes cardiotoxicity (39). In a mouse model of DOX-induced cardiomyopathy, inhibition of ferroptosis significantly improved cardiac function and reduced mortality, which was associated with the release of free cellular iron caused by HO-1 upregulation (40). DOX treatment could downregulate GPX4 and induce ferroptosis predominantly triggered in the mitochondria (41). Another study also proved that ferroptosis was involved in DOX-treated murine hearts, and acyl-CoA thioesterase 1, an important enzyme in fatty acid metabolism, might exert antiferroptosis effect in DOX-induced cardiotoxicity (42). These studies highlight that ferroptosis played a crucial role in cardiomyopathy and might be a therapeutic target.

## Iron and Cardiovascular Disease

### Role of Iron in Atherosclerotic Cardiovascular Disease

In 1981, Jerome Sullivan first proposed a hypothesis that the increased incidence of heart disease in men and post-menopausal women compared with premenopausal women could be explained by higher levels of body iron stores (5). Based on this hypothesis, numerous epidemiologic studies have investigated the role of iron in the pathogenesis of ASCVD.

The Kuopio Ischemic Heart Disease Risk Factor Study (KIHD) carried in eastern Finnish men demonstrated firstly that a high level of stored iron, assessed by increased serum ferritin, is a risk factor for myocardial infarction (MI) (43). Iron deposition was detected in coronary plaques associated with an increase of cholesterol levels in patients with atherosclerotic lesions (44). Plaques of symptomatic patients also showed higher iron concentrations and risk of cap rupture compared with plaques of asymptomatic patients (45). Several clinical studies have revealed that iron chelation therapy is beneficial to patients with CAD (46, 47). However, a systematic review and meta-analysis containing 17 prospective studies showed that there was no significant association between serum ferritin, total iron-binding capacity, serum iron, and CAD/MI, while a significant negative association was identified between transferrin saturation and CAD/MI (48). This contradicted the hypothesis that higher body iron stores represented a possible risk factor in heart disease. It may attribute to the inconsistency of the evaluated markers of serum iron in those clinical studies, and the systematic and local iron levels cannot be accurately distinguished.

The mechanism whereby iron may stimulate atherogenesis has been intensively investigated. Plenty of studies have shown an association between iron overload and atherosclerosis (49). The pathological roles of iron in atherogenesis may largely rely on its catalytically active form to generate ROS and induce lipid-peroxidation (49). Within the atherosclerotic lesions, iron overload is presented in monocytes/macrophages, endothelial cells, vascular smooth muscle cells, and platelets that all participate in the process of atherosclerosis (50). Iron overload drives endothelial dysfunction through its pro-oxidant and proinflammatory effects in endothelial cells; and promotes proliferation, apoptosis, ROS production, and phenotypic switch in vascular smooth muscle cells (51, 52). By catalyzing the atherogenic modification of low-density lipoprotein, excess iron facilitates the conversion of macrophages into foam cells (52). A recent study has reported that iron overload also enhances glycolysis and inflammation in macrophages and exacerbates the severity of atherosclerosis (53).

During the development of atherosclerosis, lipid oxidative modification and iron deposition are well-observed in plaques. It is reasonable to speculate that ferroptosis may happen in this process. A study revealed the downregulation of GPX4 in heart tissue of MI mice using quantitative proteomic analysis (54). The exosome from human umbilical cord blood-derived mesenchymal stem cells has been reported to have cardioprotective effects on mouse models of MI by inhibiting ferroptosis through suppressing DMT1 expression (55). Iron chelation therapy using desferrioxamine (DFO) has been shown to inhibit atherosclerotic lesion development (56), suggesting that ferroptosis might participate in the process of myocardial ischemia. Therefore, targeting ferroptosis might be a precise therapy of ASCVD.

### Role of Iron in Myocardial Ischemia/Reperfusion Injury

Myocardial ischemia/reperfusion (I/R) injury is an important complication of percutaneous coronary intervention or thrombolysis for acute MI (57). The recanalization of an obstructive coronary artery is effective to restore blood flow and rescue the ischemic zone, but paradoxically, reperfusion can also cause cardiac damage and necrosis due to the massive production of ROS (58). Accumulating evidence suggests that iron overload is implicated in the pathology of myocardial I/R injury (59, 60). Early studies supported that high levels of iron were mobilized into the coronary flow following prolonged ischemia, and cardiac cytosolic iron level augmented in rat hearts subjected to I/R (61, 62). A hereditary hemochromatosis model of *HFE* gene knockout mice subjected to I/R injury showed increased iron deposition, cardiomyocyte apoptosis, and ROS production compared with wild-type mice (63). Furthermore, elevated mitochondrial iron was observed in myocardial I/R injury mice and human cardiac tissue samples with ischemic cardiomyopathy, while pharmacological reduction of mitochondrial iron *in vivo* protects against I/R damage (64).

Myocardial I/R can activate hypoxia-inducible factor-1 signaling and increase TfR1 expression to facilitate iron uptake (65); upregulation of TfR1 expression in I/R-treated rat hearts was along with increased iron content (66). These illustrate that I/R can induce iron overload. Recent investigations have provided evidence that ferroptosis is involved in I/R-induced cardiomyocyte damage, and targeting ferroptosis might be beneficial for I/R conditions (40). Mitochondria-specific overexpression of GPX4 alleviates cardiac dysfunction following I/R (32). Inhibition of glutaminolysis, a component of the GSH generation pathway, can also attenuate I/R-associated heart injury by blocking ferroptosis (67). Cyanidin-3-glucoside, a subgroup of flavonoids, exhibits a protective effect in the rat model of myocardial I/R injuries *via* inhibiting USP19/Beclin1-mediated ferroptosis (68). Thus, targeting ferroptosis can serve as a potential strategy to prevent I/R-induced myocardial injury. Considerable efforts have been performed to ascertain whether iron depletion by using iron chelators could exert cardiac protection effects. In some animal models, iron chelation therapy improves contractile function, increases cell viability, attenuates cardiac remodeling, and reduces the size of infarction after I/R injury (40, 59). However, these results were not reproduced in some experimental animals (69, 70). A potential reason for the discrepancy may be species specificity. Further studies are needed to test the potential clinical implications of this therapeutic strategy.

### Role of Iron in Heart Failure

The role of iron deficiency is highly pronounced and deeply investigated in HF patients (71). Iron deficiency occurs in about 50% of chronic HF patients and is independently associated with increased morbidity and mortality (72). There are several mechanisms to explain HF-associated iron deficiency like dietary nutritional deficiency, reduced absorption caused by gut edema or proton pump inhibitors use, and gastrointestinal blooding due to the use of antiplatelets and anticoagulant agents (72).

Iron content was significantly decreased in left ventricular tissues of human failing hearts compared to HF-free organ donors, independent of anemia (73). Moreover, cardiac iron deficiency in HF was accompanied by reduced activity of aconitase and citrate synthase and reduced expression of ROS-protective enzymes (catalase, glutathione peroxidase, and superoxide dismutase 2), indicating that myocardial iron deficiency may contribute to the exacerbation of mitochondrial dysfunction that exists in HF (73). These findings are consistent with the recent research of Hoes *et al*., who demonstrated that energy production and contractile function are reduced in iron-deficient human cardiomyocytes (73). Cardiomyocytes with genetic deletion of TfR1 developed mitochondrial dysfunction, interrupted mitophagy, and promoted the metabolic switch to the fetal-like pattern (74). Thus, iron deficiency-induced mitochondrial dysfunction may reciprocally impair cellular energy supplement and cardiac function.

Because iron deficiency is associated with the pathophysiology of HF, iron repletion seems to be a therapeutic strategy for HF patients. Three main clinical trials (FAIR-HF, EFFECT-HF, and CONFIRM-HF) have proven that intravenous iron supplementation improves the quality of life, exercise tolerance, and reduces hospitalization risk for aggravated HF (75–77). Other smaller trials strengthened this evidence (78, 79). However, there is no significant clinical benefit from oral iron preparations in the IRONOUT-HF trial (80). This may be explained by the impaired iron absorption in HF (81). Although intravenous ferric carboxymaltose for symptomatic HFrEF patients with iron deficiency has been recommended in guidelines, there are several unsolved issues about iron repletion in this field. The long-term safety of intravenous iron supplementation in HF patients remains to be determined. The methods of iron administration and the potential side effects of iron should be fully considered due to iron overload-related oxidative damage.

### Role of Iron in Calcific Vascular and Valvular Disease

Vascular and valvular calcification refers to ectopic mineralization in vessel walls and heart valve leaflets. It is an important risk factor for adverse cardiovascular events (82). Although there are differences in the morphology and structure of heart valves and vasculature, the biological characteristics of vascular and valve calcification are similar. An osteoblast-like phenotypic transition of VSMCs and valvular interstitial cells (VICs) contributes directly to ectopic calcium deposition, respectively (83, 84). Both the vascular and valvular calcifications are clinically associated with the presence of diabetes, smoking, hypertension, and dyslipidemia (84). Experimental evidence demonstrates that oxidative stress has been verified to participate in pathological vascular and valvular calcification for a long time (85–87). In this context, iron-triggering oxidative stress is rational and speculated to be involved in vascular and valvular calcification.

Valve calcification mainly occurs in aortic valves. VICs differentiate into the pathological myofibroblasts and osteoblast-like cells which promote inappropriate extracellular matrix remodeling and calcification (88). Previous studies have observed that intraleaflet hemorrhage is associated with the progression of valve calcification, and iron deposition is observed within calcific valves. Interestingly, iron deposition can also be detected in non-calcified valves, suggesting that iron deposition occurs before calcium deposition at the sites of valve calcification (89). Valvular iron accumulation was observed in human calcific aortic valves, positively correlating with the degree of calcification (90). Furthermore, differentiated VICs induced by tumor necrosis factor-α and transforming growth factor-β showed significantly decreased expression of iron-exporter FPN, and in VICs isolated from stenotic aortic valves. In the presence of ferrous sulfate, VICs expressed increased ferritin subunits and exhibited proliferation capacity (90).

Ectopic vascular calcification is generally located either in atherosclerotic intima or non-atherosclerotic tunica media (91). Intimal calcification is related to arterial obstruction and atherosclerotic plaque rupture. Medial calcification leads to vascular stiffness and elevated blood pressure and pulse pressure (92). Although vascular calcification has been noted as a degenerative aging process for decades, calcification in both intima and media layers is recognized as an actively regulated process driven partly by VSMCs (91, 93). It was reported that holo-transferrin iron could promote human aorta VSMCs calcification *via* upregulating interleukin-24 (94). Some circumstantial evidence supported the relationship between iron accumulation and atherosclerosis progression (95, 96). However, there were conflicting results that iron citrate could reduce high phosphate-induced calcium deposition in VSMCs by preventing apoptosis and inducing autophagy (97), and inhibit the osteochondrogenic shift in VSMCs (98). Moreover, ferritin heavy chain exerted inhibitory effects on vascular calcification due to ferroxidase activity and antioxidant properties (99).

### Role of Iron in Pulmonary Arterial Hypertension and Systematic Hypertension

PAH is an abnormal hemodynamic state characterized by a sustained increase in pulmonary artery pressure (≥25 mmHg) and normal pulmonary capillary wedge pressure ( ≤ 15 mmHg) in the absence of other causes of precapillary pulmonary hypertension (100). PAH is classified into idiopathic, heritable, drugs and toxins-induced, and other origins (e.g., congenital heart disease, connective tissue disease, and chronic hemolytic anemias) (101). Clinical evidence supports that iron deficiency is prevalent and correlates with reduced exercise capacity and poor outcomes in both idiopathic and heritable PAH patients (101). Intravenous iron supplementation could improve quality of life and exercise endurance capacity in PAH patients with iron deficiency in two placebo-controlled studies (102, 103). To confirm the long-term effects of iron repletion, 117 PAH patients were recruited and received placebo or intravenous iron supplementation. Eighteen-month treatment with intravenous iron supplementation brought long-term clinical benefits, namely, improved risk status and reduced PAH-associated hospitalization (104). On the other hand, oral iron appeared ineffective because of impaired gastrointestinal iron absorption caused by upregulated hepcidin (105).

The underlying pathological mechanisms of iron deficiency to the PAH may involve hypoxia, inflammation, and functional alterations of pulmonary vascular cells. Hypoxia exposure can induce vasoconstriction in pulmonary arteries, resulting in increased pulmonary artery systolic pressure (PASP). Furthermore, hypoxia-induced vasoconstriction and PASP could be augmented by iron chelation with DFO in healthy adults (106). The pulmonary hypertensive response caused by altitude-induced hypoxia could be reversed by iron infusion, reducing PASP by 6 mmHg in the sea-level residents, whereas patients with chronic mountain sickness undergoing progressive iron discharge by venesection resulted in a 25% increase in PASP (107). It is reasonable to speculate that iron deficiency, analogous to hypoxia, can increase PASP, which may partly account for the pathogenesis of PAH.

It is well-known that the pathologic hallmarks of PAH contain sustained vasoconstriction, vascular remodeling, and perivascular inflammation. Since the VSMCs in pulmonary arteries are pivotal in controlling vasoconstriction, intensive attention has been paid to deciphering the role of pulmonary arterial smooth muscle cells (PASMCs) in pulmonary vascular remodeling and hypertension. Chelation of iron *in vitro* increased the metabolic activity and proliferation of human PASMCs, while iron supplementation inhibited this process. The rats fed with an iron-deficient diet developed pulmonary vascular remodeling and hemodynamic changes similar to PAH patients, which can be reversed by iron supplementation (108). Systemic iron homeostasis is controlled by FPN and its antagonist peptide hepcidin. Hepcidin treatment caused cellular iron accumulation by internalizing FPN in human PASMCs (109). In the mice expressing hepcidin-resistant isoform fpnC326Y, specific iron deficiency of PASMCs is sufficient to develop pulmonary hypertension, which was associated with markedly increased endothelin-1 (110). These results highlight the importance of intracellular iron deficiency, other than systematic iron deficiency, in the pathogenesis of PAH.

Different from pulmonary pressure, systemic blood pressure seems to be positively associated with iron markers. Two cross-sectional studies in Korea reported that serum ferritin was positively associated with the prevalence of hypertension (111, 112). Moreover, in a large-scale longitudinal study of the Chinese population, hemoglobin and transferrin levels were positively correlated with the risk of blood pressure and incident hypertension (113). Hypertensive patients with iron overload were accompanied by sympathetic overactivation but not the parasympathetic component of cardiovascular autonomic function (114). In experimental animals, dietary iron restriction attenuated cardiovascular hypertrophy, fibrosis, and inflammation in hypertensive Dahl salt-sensitive rats (115). The dietary iron restriction also prevented the development of hypertension and renal fibrosis in aldosterone/salt-induced hypertensive mice (116). These data suggested that dysregulation of iron metabolism may be an important independent risk factor for hypertension. However, detailed mechanistic information is lacking for the role of iron in systemic hypertension.

### Role of Iron in Arrhythmogenesis

Iron overload in the heart can lead to a gradual deterioration in both cardiac mechanical function and electrical activity. Chronic iron overload has been demonstrated to induce prolonged PR-interval, heart block, and atrial fibrillation in mice (117). Abnormal electrocardiograms including prolonged PR-intervals and QRS-intervals were also observed in isolated hearts of iron-treated gerbils (118). Long-term effects of iron overload resulted in frequent arrhythmias in gerbils *in vivo*, including premature ventricular contractions and supraventricular/ventricular tachycardia (119). However, arrhythmias did not occur in gerbils and guinea pigs receiving iron overload treatment despite significantly increased cardiac and hepatic iron concentrations (120, 121). The molecular mechanisms of iron-induced arrhythmias remain elusive. Many *in vitro* studies performed in isolated cardiomyocytes have verified that free iron can directly interact and interfere with a variety of ion channels of cardiomyocytes including the L-type calcium channel, the ryanodine-sensitive calcium channel, voltage-gated sodium channel, and delayed rectifier potassium channel (13, 122). Furthermore, excessive ROS production induced by iron overload could trigger the mitochondrial inner membrane anion channel opening, resulting in mitochondrial depolarization for the cytoplasmic anion efflux, which may be one of the reasons for arrhythmias (123, 124).

As for clinical studies, the incidence of arrhythmias associated with iron overload has been well-described in β-thalassemia and hereditary hemochromatosis (125). Moreover, patients with severe thalassemia and hemochromatosis could develop HF simultaneously. Iron toxicity may contribute to cardiac structural remodeling, which disturbs cardiac electrophysiological conduction. Thus, the arrhythmias occurrence induced by iron overload is confounded by the presence of HF and may not reflect the single effect of iron overload on arrhythmias. Despite limited information regarding arrhythmias occurrence with iron overload before the development of HF, a study has demonstrated that arrhythmias were significantly increased as myocardial iron deposition in patients with β-thalassemia and preserved left ventricular systolic function (126), which suggested the independent arrhythmogenic effect of iron toxicity to some extent. In addition, cardiac arrhythmias have been reported to be ameliorated by chelation in patients with iron load, which highlights iron toxicity is associated with cardiac arrhythmias.

## Conclusion and Future Directions

**Figure 3:**
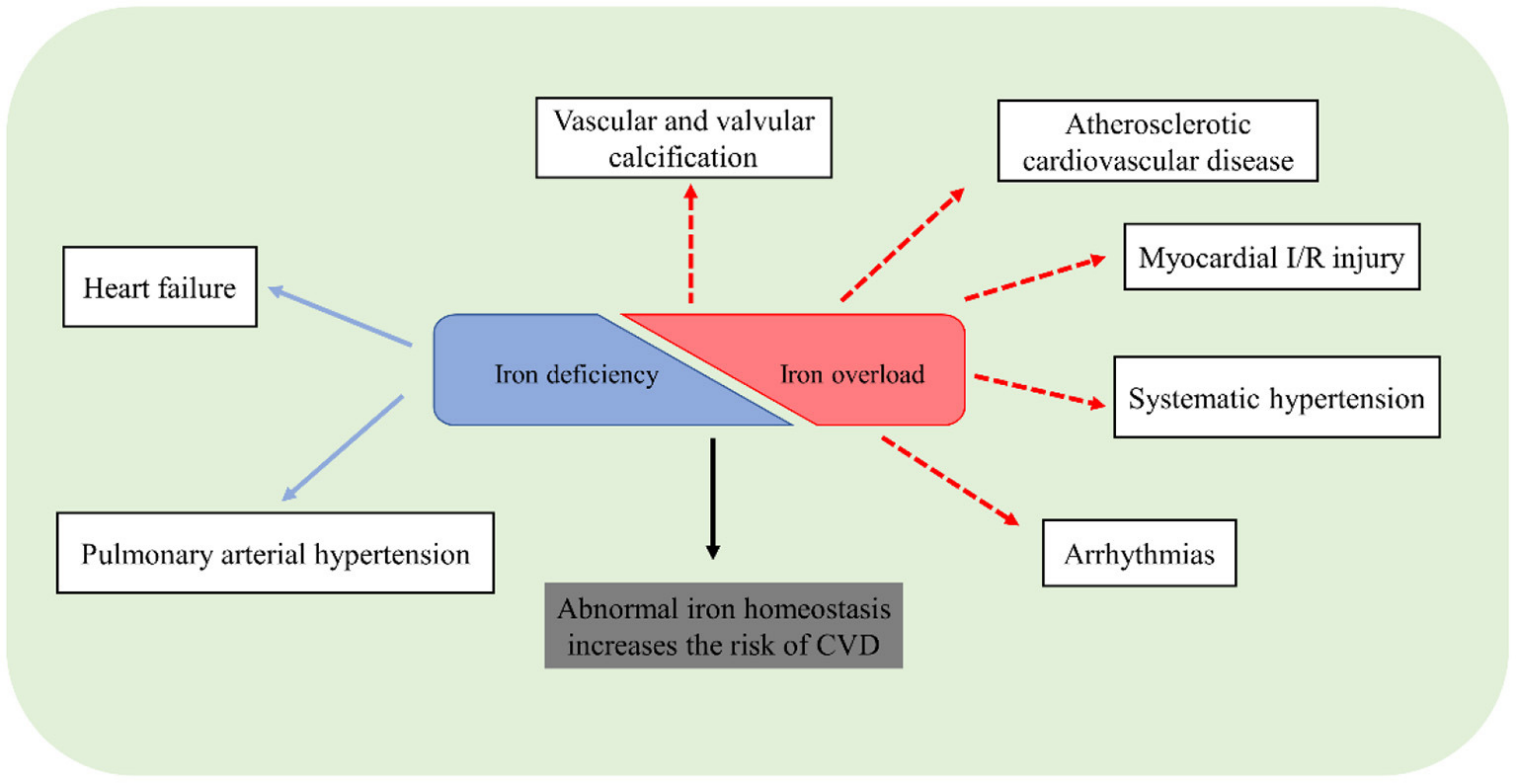
Potential association of CVD and abnormal iron homeostasis.

Iron is an indispensable micronutrient for basic biological processes. Dysregulation of iron homeostasis, inappropriate iron overload or deficiency, is harmful to a living organism. Although the understanding of the role of iron in the cardiovascular system has been advanced considerably in recent years, there remains unclear in some issues. Traditional methods cannot exactly reflect iron distribution and metabolism, especially in different tissues. Application of novel instruments or methods to measure iron, like T2 star (T2^*^) cardiac magnetic resonance imaging, is important to identify the pathophysiological roles of iron. Although iron repletion has been employed for the treatment of HF or PAH patients with iron deficiency, future studies are still necessary to pay more attention to the clinical significance of iron status and figure out the exact association between iron homeostasis and CVD (Figure 3). Ferroptosis is closely associated with the pathogenesis of CVD including cardiomyopathy, ASCVD, and myocardial I/R injury. However, the mechanisms of ferroptosis in the heart and vasculature remain elusive. The safety and efficacy of iron chelation to treat ferroptosis-related CVD require further verification.

## Author Contributions

XZ and SL: writing and editing. XZ: funding acquisition. Both authors contributed to the article and approved the submitted version.

## Funding

This study received financial support from the Scientific Research Project of Hunan Science and Technology Department (No. 2018SK52510).

## Conflict of Interest

The authors declare that the research was conducted in the absence of any commercial or financial relationships that could be construed as a potential conflict of interest.

## Publisher's Note

All claims expressed in this article are solely those of the authors and do not necessarily represent those of their affiliated organizations, or those of the publisher, the editors and the reviewers. Any product that may be evaluated in this article, or claim that may be made by its manufacturer, is not guaranteed or endorsed by the publisher.
